# A Preparatory Virtual Reality Experience Reduces Anxiety before Surgery in Gynecologic Oncology Patients: A Randomized Controlled Trial

**DOI:** 10.3390/cancers16101913

**Published:** 2024-05-17

**Authors:** Bernd C. Schmid, Dominic Marsland, Eilish Jacobs, Günther A. Rezniczek

**Affiliations:** 1 Department of Gynaecological Oncology, Gold Coast University Hospital, 1 Hospital Blvd, Southport, QLD 4215, Australia; 2 Department of Gynecology and Obstetrics, Ruhr-Universität Bochum, Marien Hospital Herne, Hölkeskampring 40, 44625 Herne, Germany

**Keywords:** virtual reality, anxiety, gynecological oncology surgery, perioperative care, randomized controlled trial

## Abstract

**Simple Summary:**

This study investigated whether using virtual reality (VR) could help reduce anxiety in women undergoing gynecological cancer surgery. Participants were split into two groups: one receiving VR therapy alongside usual care and the other receiving only usual care. Results showed that VR significantly decreased anxiety levels before surgery compared to the control group. This suggests that VR could be a valuable tool in preparing patients for surgery, potentially improving their experience and outcomes. Further research is needed to explore VR’s benefits in other types of surgery and its long-term effects on patient recovery.

**Abstract:**

Perioperative anxiety is common among patients undergoing surgery, potentially leading to negative outcomes. Immersive virtual reality (VR) has shown promise in reducing anxiety in various clinical settings. This study aimed to evaluate the effectiveness of VR in reducing perioperative anxiety in patients undergoing gynecological oncology surgery and was conducted as a single-center, double-arm, single-blinded randomized controlled trial at the Gold Coast University Hospital, Queensland, Australia. Participants were randomized into the VR intervention + care as usual (CAU) group (n = 39) and the CAU group (n = 41). Anxiety scores were assessed using a six-tier visual facial anxiety scale at baseline, after the intervention/CAU on the same day, and, several days up to weeks later, immediately before surgery. There was no significant difference in baseline anxiety scores, type of operation, or suspected cancer between the two groups. The VR intervention significantly reduced anxiety scores from baseline to preoperative assessment (*p* < 0.001). The median anxiety score in the VR intervention group decreased from 3 (interquartile range 2 to 5) at baseline to 2 (2 to 3) prior to surgery, while the control group’s scores were 4 (2 to 5) and 4 (3 to 5), respectively. Multivariate analysis showed that group assignment was the sole outcome predictor, not age, type of procedure, or the time elapsed until surgery. Thus, VR exposure was effective in reducing perioperative anxiety in patients undergoing gynecological oncology surgery. The use of VR as a preparation tool may improve patient experience and contribute to better surgical outcomes, warranting further research into exploring the potential benefits of VR in other surgical specialties and its long-term impact on patient recovery.

## 1. Introduction

Anxiety is common in cancer patients undergoing elective surgery and is one of the most challenging aspects of the perioperative period [1,2]. The incidence of preoperative anxiety is significantly higher in females than in males, making this problem particularly relevant in gynecological oncology [1,3]. As surgeons, we may overlook that the environment we work in can be intimidating and foreign to patients [4]. Matthias et al. have shown that patients who have never undergone surgery are one of the most at-risk groups, and anxiety can cause both a physiological and psychological response that can manifest itself in increasing intraoperative anesthetic requirements and prolonged postoperative recovery [5]. This stress is associated with prolonged hospital admission, the need for anxiolytics, and can be associated with takotsubo cardiomyopathy, a form of stress-induced cardiomyopathy that can occur when a person experiences severe emotional or physical stress or anxiety [6,7].

The reasons for surgical anxiety and fear of the procedure itself can vary. It may be due to the fear of the unknown or a bad experience with previous surgeries or fear of the outcome of the surgery [8]. There is also fear of the risk of death and cancer itself, which can be higher compared to surgery for benign conditions. Other reasons for perioperative anxiety include general health, leaving family, uncertainty, anesthesia, postoperative discomfort, hospital environment, and injections [3]. In psychology, in vivo exposure is the gold standard for phobia treatment. In recent years, immersive virtual reality (VR) exposure has been found to be as efficacious as in vivo exposure in the treatment of phobias [9,10]. Virtual reality implies a complete immersion experience that shuts out the physical world, and users can be transported into different environments. So far, VR has been mainly used to distract from and treat fear and anxiety, while the effect of VR exposure as a preparation tool for medical procedures is less commonly studied [11,12,13,14].

The aim of this randomized controlled trial is to familiarize patients with the perioperative suite environment using VR technology and to evaluate whether this approach can reduce preoperative anxiety. By providing virtual exposure, this study seeks to offer a more effective and comprehensive approach to managing anxiety in surgical patients, leading to improved surgical outcomes and better patient experiences.

## 2. Materials and Methods

### 2.1. Design

This single-center, double-arm, single-blinded randomized controlled trial (RCT) was conducted at the Gold Coast University Hospital (GCUH) in Queensland, Australia. The study was carried out over a 30-month period from May 2019 to December 2021.

### 2.2. Participants

Eligible participants were consecutively recruited and randomized into two groups. Participants included patients undergoing surgery at GCUH who were seen in the Gynecological Oncology outpatient clinic and scheduled for surgery. Patients presented with either a confirmed diagnosis of cancer, a high suspicion of malignancy, BRCA1/2 gene mutations, or were referred to the gynecological oncology department due to the complexity of the required surgical intervention. Exclusion criteria were as follows: patient’s age < 18 years, inability to provide informed consent, non-English speaking, and having undergone any procedure in the GCUH theater within the previous five years.

### 2.3. Procedure

**Figure 1:**
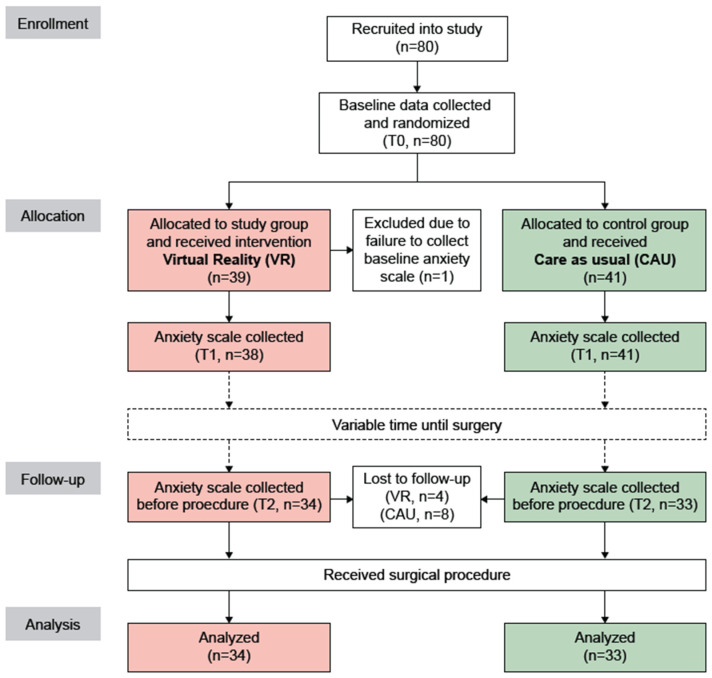
Study flow diagram. VR, virtual reality; CAU, care as usual.

Patients were informed about the study by the Gynecological Oncology clinical nurse consultant during an outpatient visit, after they had consulted with their treating doctor. Written informed consent was obtained and baseline anxiety was assessed using the assessment tool (see below) prior to randomization (baseline, time point T0). For evaluating secondary outcome measures, specific patient characteristics, such as age, type of procedure (Laparotomy/Laparoscopy/Other), and type of suspected cancer or anatomical site of pathology (ovarian, uterine, cervical, vulvar, benign) were collected. Subsequently, patients were allocated to either the VR group, in which they received the VR intervention alongside care as usual (CAU), or the control group, where patients received CAU. A second administration of the assessment tool was performed immediately afterwards (T1). The final assessment was completed by participants in the pre-anesthetic bay directly before surgery and prior to any anesthetic pre-medication (T2). The time interval between the second (T1) and third anxiety assessment (T2) was median 33 (interquartile range [IQR] 16.5 to 51) days (min 4, max 190, mean 42.8 days). Figure 1 shows the study flow diagram.

### 2.4. Virtual Reality Intervention

The VR tool incorporated elements of the real-world environment at the GCUH, featuring the pre-operative admission suite, pre-anesthetic bay, operating theatre, postoperative recovery room, and medical staff. The immersive VR experience was delivered using a VR head-mounted display (Oculus Go^®^ from Meta Reality Labs, Menlo Park, CA, USA). The intervention content presented a 360-degree, 3-dimensional video recording of the aforementioned locations. The video was produced by the authors using a Vuze 3D 360 4K VR Camera (Humaneyes Technologies Ltd., Neve Ilan, Israel) and DaVinci Resolve 15 video editing software (Blackmagic Design, Melbourne, Australia). The VR video had a duration of 3 min and 34 s (Video S1, see Supplementary Materials).

### 2.5. Assessment Tool

**Figure 2:**
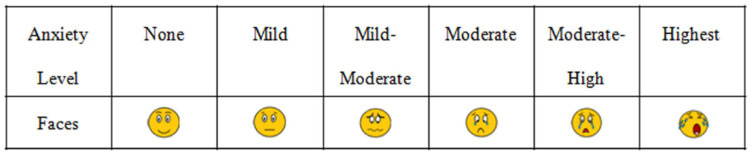
Assessment instrument; from Figure 4 in Cao et al. [15].

A novel six-tier visual facial anxiety scale for assessing preoperative fear was utilized as previously described by X. Cao (Figure 2) [15]. This visual analogue scale correlated well with Spielberger’s State-Trait Anxiety Inventory questionnaire score [8].

### 2.6. Statistics

Sample size calculation was based on a power of 80% and a significance level α of 0.05, assuming a clinically relevant difference in the anxiety scale of at least one “face” (i.e., at least 1 point on the 6-item scale) between the two groups, and a within-group standard deviation of 1.6 (effect size: 0.625). Using G power 3.1.9.2 and based on a two-sided Wilcoxon-Mann-Whitney test, it was determined that the total required recruitment number was 34 patients per group for a 1:1 group allocation [16]. Thus, assuming a drop out/lost-to-follow-up rate of up to 15%, 80 patients (40 per group) were to be recruited. A person unrelated to this study used a computerized randomization tool to generate an allocation list (block size two/four, randomization ratio one to one) and inserted paper slips with the group allocations into consecutively numbered, opaque envelopes which were opened, in order, at the time of randomization. Statistical analysis was performed in SigmaPlot 14.5 (Systat Software Inc., San Jose, CA, USA). Descriptive statistics were reported using means and SD for normally distributed data and medians and interquartile ranges (IQR) for data not meeting this assumption (Shapiro-Wilk test). Since the anxiety scale data did not follow a normal distribution, non-parametric tests were used to perform inter- and intra-group comparisons. Between-group differences at the various time points were assessed with the Mann-Whitney rank sum test; within-group differences across timepoints were assessed with the Wilcoxon signed rank test. The chi-square test was used to assess between group categorical data (intervention type, type of suspected cancer). Multiple regression analysis was performed with the differences in anxiety scores between T0 and T2 (ΔT2 − T0) as the dependent variable and age (continuous), days between T0/T1 and T2 (continuous), type of procedure (laparoscopy = 1, laparotomy = 2, other = 3), and use of VR intervention (no = 0, yes = 1) as independent variables. All *p*-values were two-tailed and *p* < 0.05 was considered statistically significant.

## 3. Results

Figure 1 shows a flow diagram of the study. Eighty patients were recruited and allocated to the VR group (n = 39) and CAU group (n = 41). One patient in the VR group was excluded after T0, because the baseline anxiety scale was not administered before randomization. Twelve patients (VR, n = 4; CAU, n = 8) were lost-to-follow-up. Thus, a total of 67 patients (VR, n = 34; CAU, n = 33) were included in the final analysis.

Patient characteristics are shown in Table 1. The groups were well balanced regarding age, suspected cancer, and procedure type. At baseline (T0), there was no significant difference in anxiety scores between the study groups (VR: median 3, IQR 2–5; CAU: median 4, IQR 2–5; *p* = 0.783; see Table 2).

**Figure 3:**
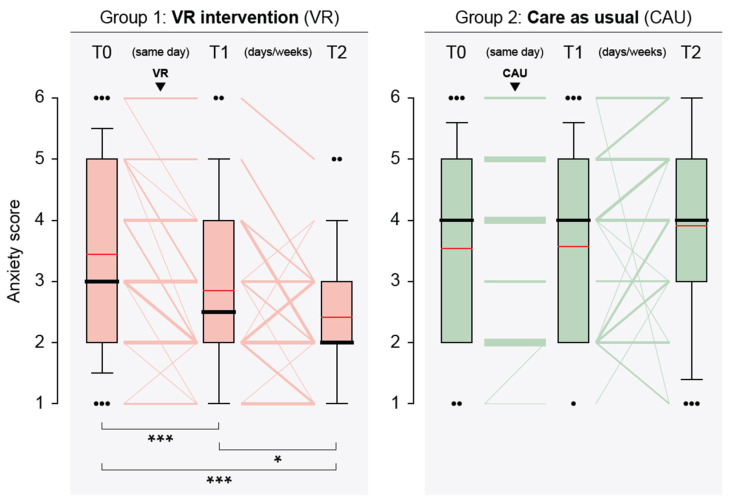
Graphical representation of anxiety levels of patients in the virtual reality intervention (VR) and care as usual (CAU) groups at the assessment timepoints (T0, baseline; T1, after VR/CAU (same day as T0); T2, before surgical procedure, days or weeks after T0/T1). Box plots: boundaries of the boxes, 25th/75th percentiles; thick horizontal lines, medians; red horizontal lines, means; whiskers, 10th and 90th percentiles; dots, outliers (when >3 outliers, only one dot is shown with the number of outliers given in parenthesis). Line plots: line thickness is proportional to the number of subjects with the same path. A statistically significant difference of anxiety scores between time points (indicated by brackets) is shown at the bottom (Wilcoxon signed rank test): ***, *p* < 0.001; *, *p* < 0.5.

The primary aim was to determine if the virtual reality (VR) intervention was effective in reducing perioperative anxiety in patients undergoing surgery. In case of the control group, anxiety scores remained unchanged at T1 (compared with T0; ΔT1 − T0 = median 0, IQR 0–0), while a significant decrease was observed for the VR group (ΔT1 − T0 = median 0 (−1–0), *p* < 0.001), demonstrating that VR was effective in reducing anxiety immediately following the intervention. Prior to the operation (T2; median 35, IQR 15–53 days after T0/T1), the VR group (median 2, IQR 2–3) continued to display significantly reduced anxiety compared to the control group (median 4, IQR 3–5), *p* < 0.001. This suggests that the VR intervention had a lasting impact on reducing anxiety even after prolonged time. In fact, the further decrease in anxiety score in the VR intervention group from T1 to T2 was statistically significant (*p* = 0.026; Table 2 and Figure 3). In contrast, anxiety scores tended to go up in the control group, but this difference was not statistically significant.

Multivariate analysis showed that the VR intervention was the sole predictor of anxiety reduction. Age, procedure type, and time span between original (T0) and immediate pre-surgical (T2) anxiety assessment had no impact (Table 3).

## 4. Discussion

The analysis of the data obtained in this randomized controlled trial demonstrates that the VR intervention was effective in significantly reducing perioperative anxiety in patients undergoing surgery. The VR intervention not only reduced anxiety immediately after its administration but also maintained the reduction in fear up until the time of surgery. The time spans between VR intervention and surgery, which varied considerably due to the different indications for surgery, had no influence on the outcome, and remarkably, anxiety scores were found to be significantly lower after this interval compared to those measured immediately after the intervention. This supports the potential use of VR as a tool for reducing perioperative anxiety in clinical practice.

The observation that the CAU group exhibited a trend towards increased anxiety directly prior to surgery compared to baseline, although not statistically significant, can be attributed to several factors: the anticipation of the surgery itself, fear of the unknown, concerns about the outcome, as well as fear of pain and discomfort. The hospital setting itself can be a source of stress and anxiety for many patients because they are confronted with an unfamiliar environment, the presence of medical equipment, seeing other patients, and the anticipation of entering the operating theatre. In its extreme form it is defined as nosocomephobia [17].

Our results are consistent with previous research highlighting the potential benefits of VR in medical settings, particularly in relation to reducing anxiety in patients undergoing various surgical procedures [12,18,19]. The immersive nature of the VR experience allows patients to familiarize themselves with the surgical environment and process, which may contribute to the observed reduction in anxiety [20,21]. One possible explanation for the effectiveness of VR in reducing anxiety is the ability to provide patients with a sense of control and predictability, which can alleviate the uncertainty associated with surgery [22], lessen anticipatory anxiety, preventing rumination and negative thought patterns, and promote a more positive mindset going into surgery [23].

In line with this, it is not surprising that some studies examining the use of VR for reducing pain during surgical interventions, such as biopsies, did not find VR to be beneficial [24]. In such a setting, the factors described above are not at play, and VR’s mode of action can merely be distraction, which may or may not work and might even be counterproductive, although varying results have been described [25,26,27].

When using VR, the possibility of cybersickness, which is like motion sickness, must be considered as a potential adverse effect [28]. Symptoms such as nausea, headaches, and dizziness may occur with the utilization of a VR headset [29]. In our study, however, none of the patients reported experiencing any of these symptoms following their VR experience. Furthermore, before VR is considered for a patient, a history of cyberaddiction should be excluded [30].

Aside from these caveats, should a healthcare service consider adopting VR to provide experiences similar to the one used here, it is important to note that the most significant expenditure would involve the production of the VR video itself. In the case of the study presented here, the technical expertise and equipment necessary for producing the 360-degree VR video were supplied by the first author. The hardware required for displaying the videos to patients, on the other hand, is comparatively cheap (<300 USD per VR goggle), and once established, it is straightforward to operate and maintain. The strength of our study lies in its robust design, including a single-blinded randomized controlled trial, a well-defined patient population, and the use of a validated assessment tool for measuring preoperative anxiety. Furthermore, our study focused on gynecological oncology patients, a population that may experience heightened anxiety due to the potentially life-altering nature of their surgeries and disease. Our study has limitations that should be acknowledged: the sample size was relatively small, and the study was conducted at a single center, which may limit the generalizability of the findings to other surgical populations and healthcare settings. In addition, the study faced challenges due to COVID-19 restrictions, which particularly affected the use of VR goggles, as these face-worn devices caused concerns regarding viral transmission.

Future research should aim to replicate these findings in larger and more diverse patient populations and investigate the long-term effects of (pre-surgical) VR interventions on postoperative outcomes, such as pain management, recovery time, and patient satisfaction. Moreover, exploring the cost-effectiveness of implementing VR interventions in routine surgical care may provide valuable insights for healthcare providers and policymakers.

## 5. Conclusions

Our study demonstrates the potential of VR as an effective tool in reducing perioperative anxiety in patients undergoing gynecological oncology surgery. By incorporating VR technology into clinical practice, healthcare providers may be able to improve patient experiences, reduce anxiety, and enhance overall surgical outcomes.

## Figures and Tables

**Table 1:** Patient characteristics.

Characteristic	Overall	VR Intervention Group 1	Care as Usual Group 2
Number of patients	67	34 (50.7%) ^1^	33 (49.3%) ^1^
Age, y	57.0 ± 13.9 [5]	54.0 ± 15.4 [3]	60.0 ± 11.7 [2]
Suspected cancer	[4]	[2]	[2]
Ovarian	26 (41.3%)	14 (43.8%)	12 (38.7%)
Uterine	26 (41.3%)	12 (37.5%)	14 (45.2%)
Vulva	6 (9.5%)	2 (6.3%)	4 (12.9%)
Cervical	2 (3.2%)	1 (3.1%)	1 (3.2%)
Benign	3 (4.8%)	3 (9.4%)	0
Procedure type	[5]	[2]	[3]
Laparoscopy	37 (59.7%)	17 (53.1%)	20 (66.7%)
Laparotomy	16 (25.8%)	11 (34.4%)	5 (16.7%)
Other	9 (14.5%)	4 (12.5%)	5 (16.7%)

Values are counts (percentage proportions; within columns unless noted otherwise) or means (standard deviations). Numbers in square brackets indicate the number of missing values. ^1^ Percentage across row.

**Table 2:** Outcomes.

Outcome	Overall (n = 67)	VR Intervention Group 1 (n = 34)	Care as Usual Group 2 (n = 33)	*p* ^3^
Outpatient visit				
Baseline anxiety ^1^ (T0)	4 (2–5)	3 (2–5)	4 (2–5)	0.783
Anxiety ^1^ after VR/CAU (T1)	3 (2–4)	2.5 (2–4)	4 (2–5)	**0.043**
*p* ^2^ T1 vs. T0	**0.002**	**<0.001**	>0.999	
ΔT1 − T0	0 (0–0)	0 (−1–0)	0 (0–0)	**<0.001**
Time interval				
Time until procedure, d	35 (15–53)	34 (14–65.75)	35 (16.5–45.5)	0.758
Day of surgery				
Anxiety ^1^ before surgery (T2)	3 (2–4)	2 (2–3)	4 (3–5)	**<0.001**
*p* ^2^ T2 vs. T0	0.085	**<0.001**	0.064	
*p* ^2^ T2 vs. T1	0.763	**0.026**	0.076	
ΔT2 − T0	0 (−1–1)	−1 (−2–0)	0 (0–1)	**<0.001**
ΔT2 − T1	0 (−1–1)	0 (−1–0)	0 (0–1)	**0.004**

Values are counts or medians (interquartile ranges). Numbers in brackets indicate the number of missing values. VR, virtual reality; CAU, care as usual; d, days. Bold indicates statistical significance (*p* < 0.05). ^1^ Visual facial anxiety scale (1 = smiling face, anxiety level of “none”, 6 = crying face, anxiety level of “highest”). ^2^ *p* values for the comparison of anxiety at time points within groups (Wilcoxon signed rank test). ^3^ *p* values for the comparison of group 1 with group 2 (Mann-Whitney U test).

**Table 3:** Multiple linear regression analyses.

Independent Variables	Dependent Variable: ΔT2 − T0 [8] *p*
Age [5]	0.185
Time until surgical procedure	0.438
Type of procedure [5]	0.335
Virtual reality intervention	**0.001**

ΔT2 − T0, difference of anxiety scores measured before surgery (T2) and at baseline (T0). Bold indicates statistical significance (*p* < 0.05). Number of observations: n = 59. Numbers in square brackets indicate the numbers of missing observations. Independent variables were as follows: age (continuous, in years); time until surgical procedure (continuous, in days); type of procedure (encoded as laparoscopy = 1, laparotomy = 2, other = 3); and virtual reality intervention (encoded as 1 = yes, 0 = no).

## Data Availability

Available upon reasonable request.

## Supplementary Materials

The following supporting information can be downloaded at: https://doi.org/10.5281/zenodo.10997439 (accessed on 19 April 2024), Video S1: The VR content shown to participants in the VR group.
